# Spatial distribution, pollution level and human health risk assessment of heavy metals in urban street dust at neighbourhood scale

**DOI:** 10.1007/s00484-024-02729-y

**Published:** 2024-07-02

**Authors:** Oznur Isinkaralar, Kaan Isinkaralar, Tuyet Nam Thi Nguyen

**Affiliations:** 1 Department of Landscape Architecture, Faculty of Engineering and Architecture, Kastamonu University, 37150 Kastamonu, Türkiye; 2 Department of Environmental Engineering, Faculty of Engineering and Architecture, Kastamonu University, 37150 Kastamonu, Türkiye; 3 Faculty of Environment, Saigon University, 273 An Duong Vuong Street, District 5, Ho Chi Minh City, Vietnam

**Keywords:** Ecological risks, Human exposure, Urban modeling, Land use, Environmental quality, Land management

## Abstract

Urban street dust (UStD) is a vital issue for human health and is crucial for urban sustainability. This study aims to enhance the creation of safe, affordable, and resilient cities by examining environmental contamination and health risks in urban residential areas. Specifically, it investigates the concentrations and spatial distribution of chromium (Cr), cadmium (Cd), nickel (Ni), copper (Cu), lead (Pb), and zinc (Zn) in UStD in Yenimahalle, Ankara. The mean concentrations of Zn, Cr, Pb, Cd, Ni, and Cu in UStD were 97.98, 66.88, 55.22, 52.45, 38.37, and 3.81 mg/kg, respectively. The geoaccumulation pollution index (Igeo) values for these elements were: Cd (5.12), Ni (1.61), Cr (1.21), Pb (1.13), Cu (0.78), and Zn (0.24). These indices indicate that the area is moderately polluted with Cr, Pb, and Ni, uncontaminated to moderately contaminated with Cu and Zn, and extremely polluted with Cd. The hazard index (HI) values for Cr, Cd, Ni, Cu, Pb, and Zn were below the non-carcinogenic risk threshold for adults, indicating no significant risk. However, for children, the HI values for Pb, Ni, Cd, and Zn were 3.37, 1.80, 1.25, and 1.25, respectively, suggesting a higher risk. Carcinogenic risk (RI) of Cd, Ni, and Pb was significant for both children and adults, indicating that exposure through ingestion, inhalation, and dermal contact is hazardous. The findings highlight the need for strategic mitigation measures for both natural and anthropogenic activities, providing essential insights for residents, policymakers, stakeholders, and urban planners.

## Introduction

Today, cities are essential organizations for culture, ideas, creativity, productivity, science, commercial and social advancement (Huang et al. 2020). However, this potential phenomenon is under threat from urban growth and the exponential increase in the human population (Feng et al. 2020). Forecasts predict that the urban population will climb until 2050 (United Nations 2019). The cities have been facing increased pollution, chaotic traffic, environmental degradation, unemployment, and a lack of clean, green, and relaxing public spaces for sustainable future of the world (Kaur and Pandey 2021). Local governments, especially in low- and middle-income countries, have been unable to cope with increasing environmental-social problems (Zhao et al. 2023), despite their efforts to tackle emerging environmental problems (Bibri et al. 2020). Urban planners and administrators face daily challenges in maintaining clean water, air, energy, housing, thermal comfort (Yin et al. 2023), and green spaces (Wen et al. 2024) by investing in green infrastructure. In particular, urban planners are challenged with protecting clean water, air, energy, housing, and green spaces, reorganizing urban locations, and expanding smart urbanization movements for pollution routing problems (Xiao et al. 2020). From this perspective, urban streets integrated into local planning and management are vital components of human society and are critical to the urban environment. If properly planned and managed, urban streets can make valuable contributions to the quality of urban human life and ecological systems.

The heavy metals in UStD mainly originate from the atmospheric deposition of particles re-suspended under the effect of winds and synoptic weather (Lu et al. 2022). Anthropogenic emissions include construction, municipal projects, transportation, commercial activities, and waste management, a major sink of toxic heavy metals (HMs) in UStD in cities (Voordeckers et al. 2021). To address this, it is well known that it contains some potentially toxic metals such as chrome (Cr), cadmium (Cd), nickel (Ni), copper (Cu), lead (Pb) and zinc (Zn), which hazards ecological systems due to their persistence-durability and bioaccumulation-deposition in tissues and organs, also adverse and toxicity effect to human health via three major pathways (inhalation, ingestion, and skin contact) (Sah et al. 2017). For this reason, measuring HMs in UStD is critical for understanding variations in the environmental quality of urban areas negatively influenced by inappropriate human activities. The airborne toxic metals contamination in UStD remains a significant concern and is predominantly sourced from the release of vehicular emissions and heating activities. These metals can occur with wind erosion of transportation, accumulation in various environmental settings, and disparities in industrializing and expanding cities because transport and deposition play several critical roles, affecting land degradation, climate, the environment, and public health (Aguilera et al. 2021). The impact of UStD on human health depends on both the metal concentration characteristics of the dust particles and their emitting source. Faced with these causes, toxic metals in cities are highly diverse and easy to accumulate but difficult to disperse due to atmospheric conditions (Fang et al. 2015). Large quantities of pollutants in the outdoor environment passing indoors cause damage to the ecosystem in several ways, and humans of all age groups are exposed significantly in the accumulation area. Especially toddlers (under eight years old) are more vulnerable to this risk through ingestion. Investigations of the hazards of metal pollution in urban areas can reveal unique insights into the challenges faced by cities with similar industrial profiles. Numerous studies have addressed this negative impact and underscored a connection between toxic metal contamination and health risk assessment across all age groups.

In Türkiye, metals in UstD, such as Cd, Cr, Cu, Ni, and Pb, were investigated in several areas, including Eskişehir located in western Türkiye (Isinkaralar et al. 2024a), Düzce City Center (Isinkaralar et al. 2024b), Ankara which is a capital of Türkiye (Isinkaralar et al. 2023), Dilovasi (Ulutaş 2022), Sivas (Nuhoglu et al. 2020), Meles River basin (Guven 2019), and Konya, one of the industrialized cities of Türkiye (Kariper et al. 2019). Results from these studies revealed that exposure to heavy metals, such as Cd, Cr, Ni, and Pb, could cause potential risks for human health, especially for children (Isinkaralar et al. 2023, 2024a, b). Additionally, metals in several cities of Türkiye mainly originated from transportation and residential activities (Ulutaş 2022) as well as industrial production (Guven 2019; Kariper et al. 2019).

Yenimahalle has one of the most diverse production and processing sectors in Ankara and perhaps even Türkiye. However, metal pollution and its effect on human health have not been investigated in Yenimahalle. Therefore, in this study, ecological and human health risks associated with potential toxic metal (Cr, Cd, Ni, Cu, Pb, and Zn) concentrations and the distribution of their sources in Yenimahalle were investigated, considering densely populated settlements and high land use diversity. Studying pollution in similar urban and industrial contexts will provide global insights into pollution-related health and environmental repercussions. The findings may have policy implications for urban planning, industrial codes, and public health interventions in developing countries facing similar industrial and urbanization challenges top-down strategy. They may help develop solutions to limit metal pollution and its consequences.

## Material and methods

### Study area

**Fig. 1:**
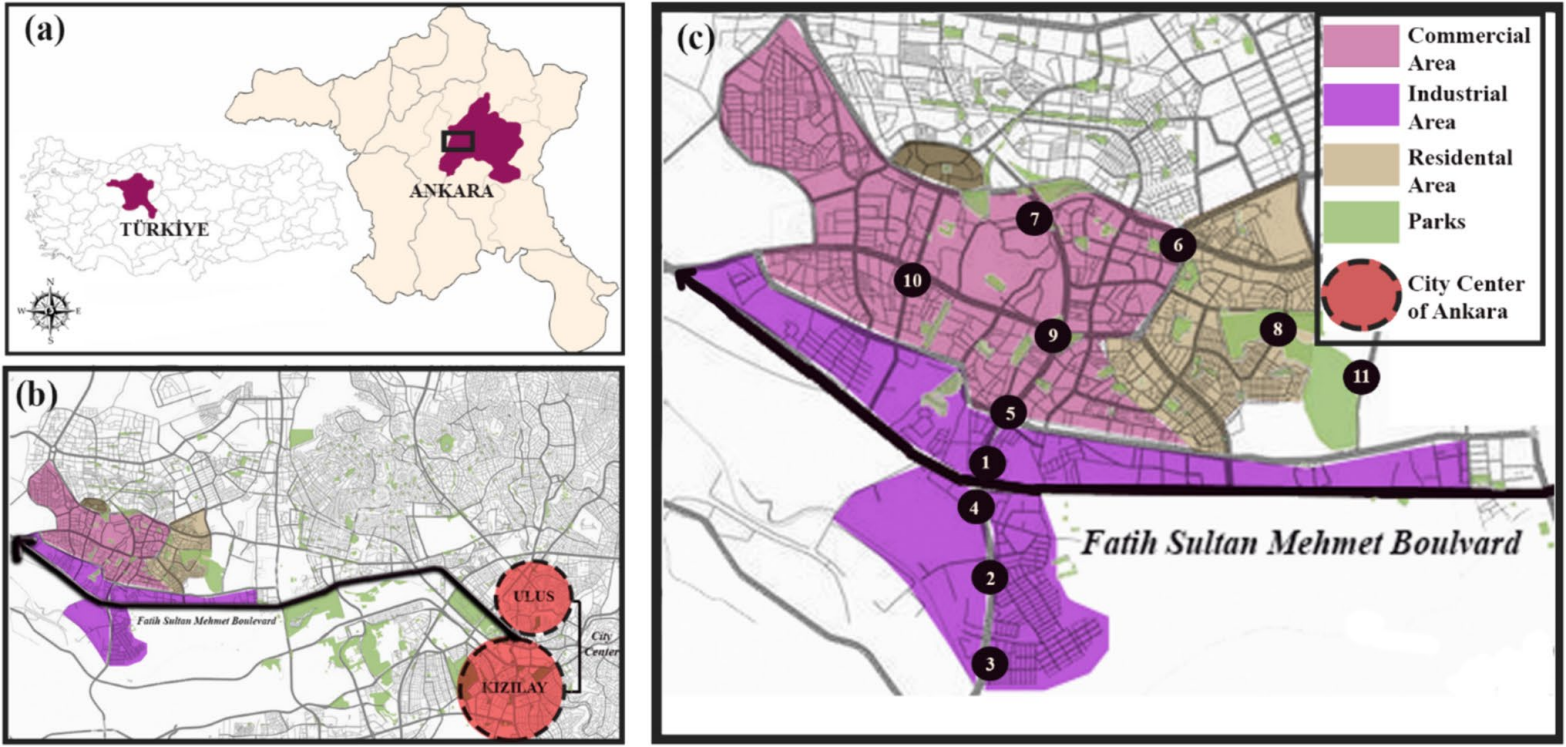
Map of locations: **a** Ankara in Türkiye; **b** sampling area relative to the city center; **c** the sampling points

Yenimahalle is situated in Ankara, Türkiye. It covers approximately 436 km^2^ and has a population of 695.395 inhabitants (Turkish Statistical Institute 2022) as shown in Fig. 1. It is densely populated because it has the Ankara industry's pioneering industrial zone, OSTİM. Industrial activities, including more than 6,200 companies and more than 60,000 workers, are active in 17 different sectors and 139 lines of business. In this area, the manufacturing sectors are metal processing and smelting (62%), plastics-rubber (20%), electricity (8%), and chemical production (4%), so their contribution to atmospheric pollution is significant due to toxic and metal emissions. The region's climate is strongly continental and highly vulnerable to wind erosion/dust emissions, and average rainfall is often poor in both summer and fall.

Urban land patterns influence people living in built-up areas' exposure to priority pollutants produced by various sources. Urban land uses with the potential for the release and storage of pollutants were diversified in determining the sample areas. These are considered in terms of proximity distance (α) to main arteries, industry, parking areas, and settlements as shown in Table 1.

**Table 1:** Location selection criteria

Sampling number	Distance to main artery	Distance to ındustrial area	Distance to commercial areas	Distance to green areas	Population/Building density
1	H	H	L	L	−
2	M	H	L	L	−
3	L	H	L	L	−
4	H	M	L	L	−
5	H	M	M	L	−
6	M	L	L	L	+
7	M	L	M	L	+
8	L	L	Low	H	+
9	L	L	H	H	+
10	L	L	H	L	+
11	L	L	Low	L	−

Low (L) α < 200 *m, Medium* (M) 200 m < α < 500 m, High (H) α > 500 m

### Sampling and analysis

UStD samples were investigated to characterize their total toxic metal content. One hundred twenty-one samples were collected during the summer of 2023 (June, July, and August). Individual points were selected, and each sample was gathered from 11 selected spots located in areas with high traffic and population density. UStD was brushed from a 0.5 m^2^ frame in each area with a polyethylene brush (5.6 cm) and a plastic hand shovel. The samples were bagged for transport to the laboratory. All UStD samples were dried at 50 °C for 48 h, sieved with a 2 mm mesh size, and 0.5 g of each dust sample was separated. Then, the coarse contaminants were separated for Cr, Cd, Ni, Cu, Pb, and Zn analysis. The dust samples were sieved using a 2 mm mesh size and digested following the standard procedure applicable to microwave-assisted rapid multi-element extraction using the 3050B USEPA technique (US Environmental Protection Agency (USEPA) 1996). This representative sample was placed in a polytetrafluoroethylene (PTFE)-based polymer microwave vessel. To this, 10 mL aqua-regia (3:1 mixture of HCl-37% and HNO_3_-69%) was added and heated in a microwave unit for a programmed period. The vessel was then cooled, and the contents were transferred to a 50 mL centrifuge tube. The volume of the extractant is made up to 50 mL using a 10% HNO_3_ solution. Finally, after centrifuging and filtered through a Whatman filter, the analyte was detected for metal concentrations (mg·kg-1 dw) via inductively coupled plasma optical emission spectroscopy (ICP-OES) (ICP-OES, Germany).

### Statistical analysis

To determine the relationship between toxic metals in UStD and their possible origins, the Pearson correlation coefficient and cluster analysis (CA) were performed with the SPSS version 22.0 statistical software package. Its correlation coefficient measures the relative strength of the relationship between two trace metals and is the most widely accepted multivariate statistical method in applied environmental studies. The standard deviation (S.D.) was calculated and the relative standard deviation S.D. was < 5%, indicating good precision.

### Assessment of data quality

Ensuring meticulous quality control and assurance, all reagents used in the experiments were analytical grade to make sure they satisfied quality standards and delivered reliable and consistent results. To prepare the standards and solutions, deionized water was primarily employed to eliminate potential contamination from impurities in normal tap water and improve the experimental procedures' accuracy and repeatability. Tests were executed in three replicates, and the average value was presented. The limits of quantification (LOQs) were derived using the calibration curve at the lowest concentration. The LOQ of Cr, Cd, Ni, Cu, Pb, and Zn analyzed in samples was 0.1 mg·kg^−1^. For statistical analysis, concentrations under the LOQ were attributed as high quality / reliable with a value of 0.01 × LOQ.

### Ecological risk assessment

Ecological Risk estimates the probability that being exposed to physical and chemical risk factor stressors will negatively affect the ecology. Various factors that contribute to unfavorable environmental responses are highlighted in this section. Table 2 presents the geoaccumulation pollution index (*I*_*geo*_) classification, and Eq. (1) is given to compare pollution levels with background concentrations (${C}_{n}:$ metal concentration mg·kg^−1^ and ${B}_{n}$: geochemical background values mg·kg^−1^).

**Table 2:** Geoaccumulation pollution index classification

Class	*I*_*geo*_ Value	Classification
0	< 0	Unp
1	0–1	Unp to Mp
2	1–2	Mp
3	2–3	Mp to Sp
4	3–4	Sp
5	4–5	Sp to Ep
6	> 5	Ep

Unpolluted (Unp), Unpolluted to moderately polluted (Unp to Mp), Moderately polluted (Mp), Moderately to strongly polluted (Mp to Sp), Strongly polluted (Sp), Strongly polluted to extremely polluted (Sp to Ep), Extremely polluted (Ep)

1 $${I}_{geo}={Log}_{2} \left(\frac{{C}_{n}}{1.5{B}_{n}}\right)$$

Another assessment equation is the Enrichment Factor (EF) in Eq. (2) (Buat-Menard and Chesselet 1979); trace metal contamination levels can be compared to the surrounding environment. The metal amounts (MA) were also normalized for soil (S) and earth's crust (EC) using Fe as a reference metal in Eq. (2).

2 $$EF=\frac{{MA}_{S}/Fe\; for\; soil}{M{A}_{EC}/{Fe}^{\prime}\; for\; earth\; crust}$$

The *EF* value is smaller than one, which means that the element mainly comes from the crust and other natural sources, while an *EF* larger than 1 implies that it is affected by both human and natural factors. The *EFs* are categorized for enrichment level as the minimum (1–2), moderate (2–5), significant (5–20), very high (20–40), and extremely strong (> 40) (Ekwere and Edet 2021).

Ecological Risk Index (*RI*) describes the degree of contamination of each metal based on their adverse environmental risk in Eq. (3).

3 $$\begin{array}{ccc}RI: {\sum }_{i:1}^{n}PER& PER: {T}_{i}{f}_{i}& {f}_{i}: {C}_{i}/{B}_{i}\end{array}$$

Here *C*_*i*_ is the HMs concentration, and *C*_*r*_ is the reference metal concentration (mg·kg^−1^) from our experimental analysis. *PER* is the potential ecological risk (PER) factor of metal i, and *T*_*i*_ is the metal toxic factor. ƒ_i_ is the metal pollution factor of metal i, which equals the amount of metal i in the sample (*C*_*i*_) divided by its *B*_*i*_, which are reference values for metals, *C*_*i*_ is the content of metals in UStD. Classification levels were determined as low (< 150), moderate (150–300), high (300–600), severe (> 600) for *RI* and low (< 40), moderate (40–80), high (80–160), serious (160–320), severe (> 320) for *E*_*i*_.

### Human health risk assessment

The Health Risk Assessment model describes exposure to metals for both adults and children by USEPA (1989, 2001, 2007, 2011) and was calculated through the Hazard Index (*HI*) and hazard quotient (*HQ*) for non-carcinogenic risk. The average daily dose of exposure through ingestion is in Eq. (4), inhalation is in Eq. (5), and dermal is in Eq. (6).

4 $${D}_{Ing}: \frac{IngRxEFxED}{BWxAT}x {10}^{-6}$$

5 $${D}_{Inh}:C x \frac{InhRxEFxED}{PEFxBWxAT}$$

6 $${D}_{Der}:C x \frac{SLxSAxABSxEFxED}{BWxAT}x {10}^{-6}$$

Parameters of health risk assessment were used to determine some standard values for children and adults. Ingestion rate (*IngR*_*child*_, and *IngR*_*adult*_) are 200 and 100 mg·day^−1^ for (US Environmental Protection Agency (USEPA) 2001); Inhalation rate (*InhR*_*child*_, and *InghR*_*adult*_) are 9.3 and 16.3 m^3^ day^−1^ (US Environmental Protection Agency (USEPA) 2011); Particle emission factor (*EF*_child_ and *PEF*_adult_) are 1.36E + 09 m^3^·kg^−1^ (US Environmental Protection Agency (USEPA) 2001); Skin adherence factor (*SL*_*child*_ and *SL*_*adult*_) are 0.2 and 0.07 mg·cm^−2^·day^−1^ (US Environmental Protection Agency (USEPA) 2001); Exposed skin area (*SA*_*child*_ and *SA*_*adult*_) are 2800 and 5700 cm^2^ (US Environmental Protection Agency (USEPA) 2001); Dermal absorption factor (*ABS*_*child*_ and *ABS*_*adult*_) are 0.001 (US Environmental Protection Agency (USEPA) 2001); Exposure frequency (*EF*_*child*_ and *EF*_*adult*_) are 180 day·year^−1^ (Zheng et al. 2010; Hu et al. 2011); Exposure duration (*ED*_*child*_ and *ED*_*adult*_) are 6 and 24 years; Body weight (*BW*_*child*_ and *BW*_*adult*_) are 22.5 and 68.43 kg (US Environmental Protection Agency (USEPA) 2011); average exposure time (*AT*_*child*_ and *AT*_*adult*_) are 2190 and 8760 days.

7 $$HI: \left\{\left({HQ}_{Ing}\right)+\left({HQ}_{Inh}\right)+\left({HQ}_{der}\right)\right\} : \left\{\left(\frac{{D}_{Ing}}{{R}_{f}{D}_{Ing}}\right)+\left(\frac{{D}_{Inh}}{{R}_{f}{D}_{Inh}}\right)+\left(\frac{{D}_{Der}}{{R}_{f}{D}_{Der}}\right)\right\}$$

Different exposure pathways which *R*_*f*_*D*_*Ing*_*, R*_*f*_*D*_*Inh*_*,* and *R*_*f*_*D*_*Der*_ are varied, and the range value from 6.00E-05 to 1.20E-02 for the expressed reference dose in Eq. (7). The *HQ* is expressed as the ratio between the average daily dosage received through several pathways (*D*_*ing*_*, D*_*inh*_, and *D*_*der*_) and the reference dose (*RfD*) for a given toxic metal. The *HI* > 1 means probable non-carcinogenic activity of toxic metals; *HI* < 1 suggests no health risk. Also, all variables are used as a guide in the human health risk assessment model based on USEPA (1989; 2001; 2002; 2004; 2009; 2007).

The evaluation of cancerous risk by using the lifetime average daily doses (LADD) for a number of carcinogenic substances for Cr, Cd, Ni, Cu, Pb, and Zn. LADD has been calculated as follows:

8 $${LADD}_{inh}= \frac{C\times \text{EF}}{\text{ PEF}\times \text{AT}}\times \left(\frac{{InhR}_{child}\times {ED}_{child}}{{BW}_{child}}+\frac{{InhR}_{adult}\times {ED}_{adult}}{{BW}_{adult}}\right)$$

9 $$\text{Carcinogenic risk }(\text{RI})\hspace{0.17em}=\hspace{0.17em}\text{LADD}\hspace{0.17em}\times \hspace{0.17em}\text{SF }$$

where ${InhR}_{child}$ is the absorption rate; for each exposure, the cancer slope factor (SF) is multiplied by the lifetime average daily dose (LADD) (mg kg^−1^ day^−1^) to determine the estimated carcinogenic risk (Khairy et al. 2011). An RI value between 10^–6^ and 10^–4^ is regarded as a permissible risk limit (US Environmental Protection Agency (USEPA) 1996) and the levels of risk were categorized by Rapant et al. (2011) as RI is very high (> 10^–3^), high (10^–4^ -10^–3^), medium (10^–6^ -10^–4^), low (10^–6^ -10^–5^) and extremely low (< 10^–6^).

## Results and discussion

### Impact of industrial operations

Table 3 presents the descriptive statistics of Cr, Ni, Cu, Cd, Pb, and Zn concentrations, which followed the order Zn > Cr > Pb > Cd > Ni > Cu and were 97.98 (52.20–141.20) mg/kg, 66.88 (11.20–148.20) mg/kg, 55.22 (10.20–99.20) mg/kg, 52.45 (19.20–90.20) mg/kg, 38.37 (6.20–71.20) mg/kg, and 3.81 (1.39–6.80) mg/kg, respectively. Zn, Pb, Cd, and Cr concentrations were significantly higher than others and exceeded the WHO limits. The mean concentrations of Cd, Ni, Cr, and Pb exceeded the values recorded for the Upper Continental Crust. There was no considerable variation in concentrations between streets and sampling days, suggesting a higher contamination risk from metal processing activities. Yenimahalle has a unique characteristic combination of elemental compositions by industrial activities. The skewness values for Cr, Ni, Cu, and Cd were largely positive, indicating that the means were higher than the median, suggesting the presence of high pollution events and the temporal nature of the highest concentrations between sampling points. They indicate a positive skew towards low concentrations. Except for Cu, the mean concentration value for Zn, Pb, Cd, and Cr was several times higher than that of the background concentrations, indicating possible anthropogenic input of toxic metals from the wear of motor vehicle parts, the combustion of fossil fuels, and metal processing.

**Table 3:** The descriptive statistics of Cr, Ni, Cu, Cd, Pb, and Zn (mg kg^−1^) in UStD samples

Elements	Min	Max	Mean	Percentiles%			SD	CV%	Skewness	Kurtosis
				25	50	75				
Cr	11.20	148.20	66.88	33.70	60.20	91.20	35.54	53.13	0.59	−0.35
Ni	6.20	71.20	38.37	39.20	53.20	61.20	13.57	35.38	0.09	0.38
Cu	1.39	6.80	3.81	30.20	36.20	46.70	1.31	34.45	0.25	−0.37
Cd	19.20	90.20	52.45	2.99	3.50	4.80	15.99	30.49	0.30	−0.05
Pb	10.20	99.20	55.22	40.20	54.20	69.20	20.68	37.45	−0.11	−0.22
Zn	52.20	141.20	97.98	82.20	96.20	112.20	21.51	21.96	−0.06	−0.35

The findings of the literature align with the mean concentrations of HMs reported in UStD (Zheng et al. 2020; Chen et al. 2022), which could be due to toxicity variance and the extensive effect of multiple contaminants (Jiang et al. 2018; Huang et al. 2022; Dat et al. 2021). Furthermore, the average metal concentrations are relatively high when compared to the conditions reported by Sobhanardakani (2018); Zhaoyong et al. (2019), and Bartholomew et al. (2020). Data from this review are comparable to UStD reported by Abdulaziz et al. (2022) in Saudi Arabia, where the intricate interactions between HMs contamination and health risk assessment were analyzed. The given Cr concentration exceeded the excess lifetime risk-related limit of 1:10^4^, described according to the World Health Organization (WHO) as 0.0025 µg/m^3^. The study of Hashemi et al. (2020) on HMs (Zn, Cu, Pb, Cd, Cr, and Ni) in indoor dust of Bushehr showed that UStD influences indoor dust and is an increasing public health concern due to ingestion of indoor dust from source apportionment. The Pb, Cu, Zn, Ni, Cr and Cd are attributed to large vehicle traffic emissions (Bi et al. 2018; Bernardino et al. 2019); wear of engine parts and fuel and oil leakage (Dong et al. 2017); vehicle brake linings (Haynes et al. 2020), metallic parts and corrosion of automobile parts (Duong and Lee 2011); Ni plating and alloys and yellow paint on roads (Bruce et al. 2021); and erosion and wear of motor vehicle parts (Krupnova et al. 2020); corrosion of building materials and fertilizer application (Castillo-Nava et al. 2020).

### Impact of industrial activities

**Fig. 2:**
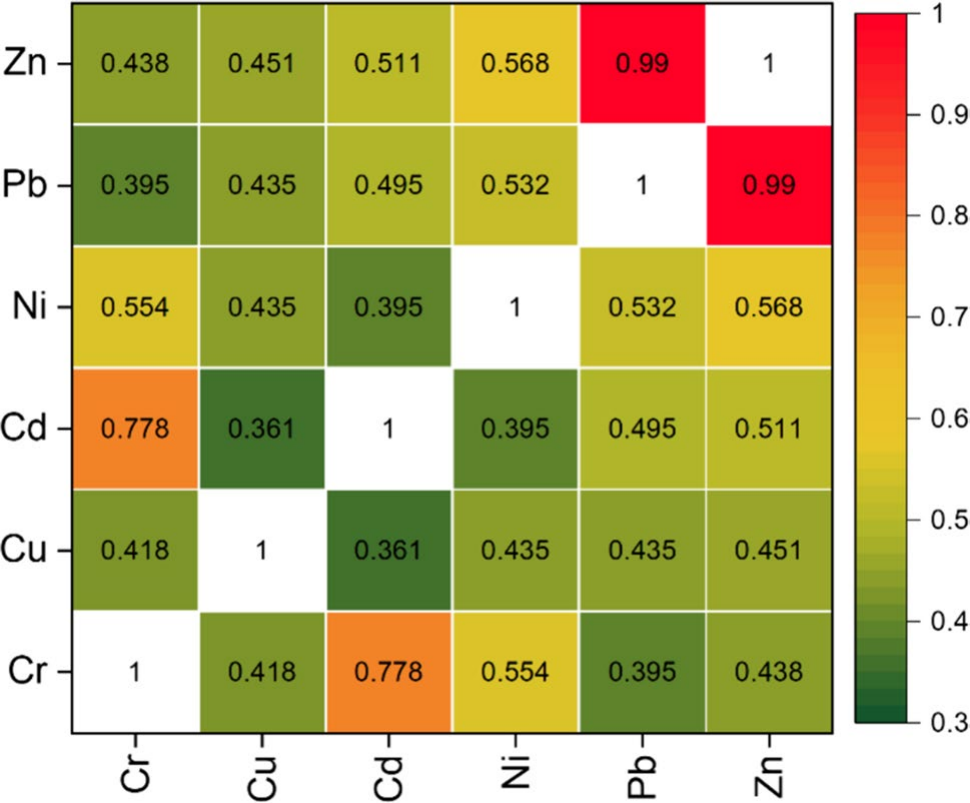
Correlation coefficients for potentially toxic metals

Correlation coefficients between metals represent common origin with potential natural and persistent anthropogenic sources. From our analysis, we infer that Cr, Ni, Cu, Pb, and Cd had significant positive correlations with each other. Zn and Pb showed significantly strong correlations with r: 0.99** because they represented industrial-related emissions. Similarly, Cd and Cr had positive correlations with r: 0.778**, whereas Cu only had a slight correlation with Cd with r: 0.361* due to Cd and Cu having different sources (Fig. 2). This suggests that Cd and Cu are partly derived from a natural source (local soil), whereas industrial operations mainly impact Ni, Cd, and Cr. A previous paper by Zhang et al. (2020) reported that the bioaccessibility of Pb and Cd could be principally ascribed to coal combustion, automobile exhaust emissions, and paint and fertilizers. According to the correlation analysis in other cities (Goudarzi et al. 2018; Nargis et al. 2022), some toxic metals are closely related to the same sources in busy areas of urban regions (Xie et al. 2019; Isinkaralar et al. 2023).

### Ecological risk

According to *I*_geo_, values were calculated to assess metal pollution in Yenimahalle, and the results are presented in Table 4. The mean *I*_geo_ values of Cr, Ni, Cu, Cd, Pb, and Zn were 1.21 (-0.09–0.17), 1.61 (0.90–1.97), 0.78 (-0.54–1.48), 5.12 (4.23–5.76), 1.13 (-0.43–1.65), and 0.24 (-0.3–0.58), respectively, in the following sequence: Cd > Ni > Cr > Pb > Cu > Zn. This indicates that the street dusts were moderately polluted with Cr, Pb, and Ni, uncontaminated to moderately contaminated with Cu and Zn, and highly polluted with Cd. Many scholars have paid more attention that the pollution degree at different sampling sites shows differences by Wei et al. (2015), Wang et al. (2018), and Han et al. (2020). This may explain why there are outliers in the pollution index values of some metals.

**Table 4:** *I*_geo_ values of potentially toxic metals in UStD

Elements	Range of *I*_geo_	Mean of *I*_geo_	Pollution levels
Cr	−0.09–0.17	1.21	Mp
Ni	0.90–1.97	1.61	Mp
Cu	−0.54–1.48	0.78	Unp to Mp
Cd	4.23–5.76	5.12	Ep
Pb	−0.43–1.65	1.13	Mp
Zn	−0.3–0.58	0.24	Unp to Mp

Moderately polluted (Mp), Unpolluted to moderately polluted (Unp to Mp), and Extremely polluted (Ep)

**Fig. 3:**
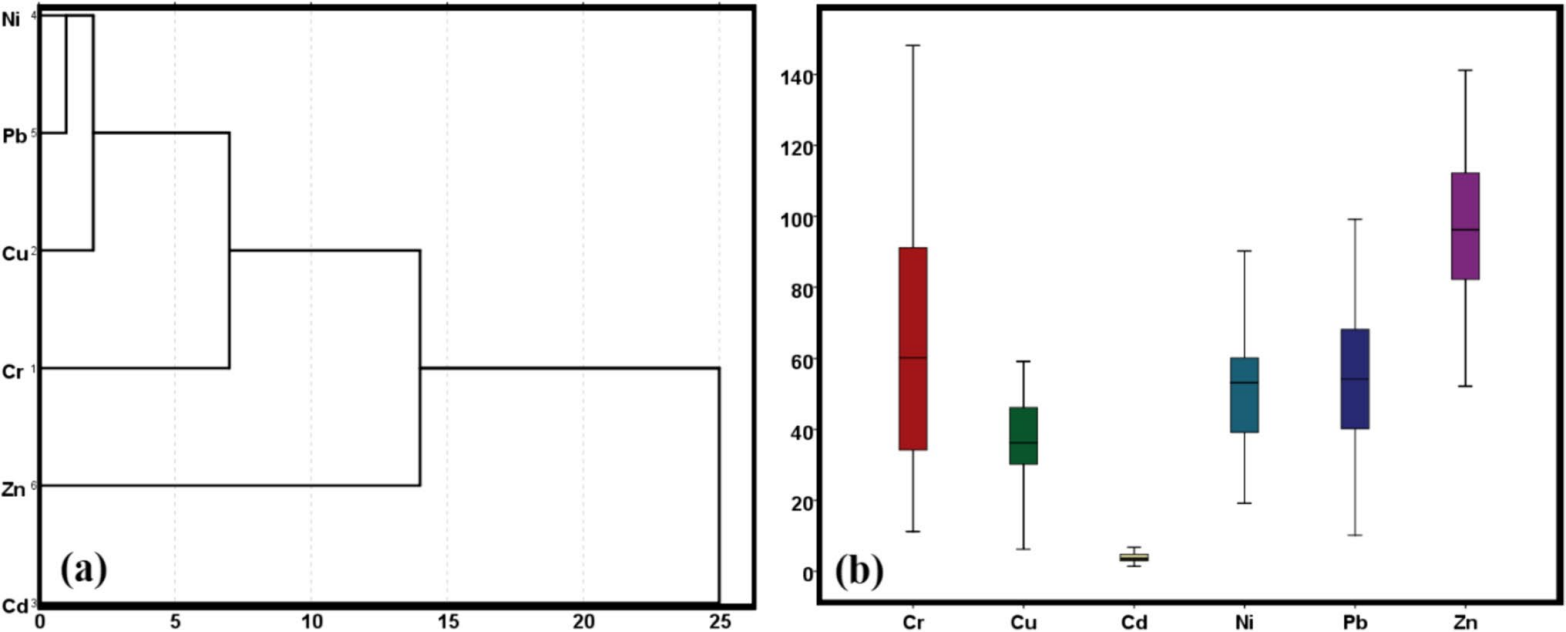
(**a**): Dendrogram showing cluster and (**b**): box plot of toxic metals concentrations in UStD

Figure 3 displays dendrogram results in four clusters: Ni-Pb, Ni-Cu, Cr-Cu, Pb, Ni, and Cd, which are fully consistent with the correlation results. However, clusters 3 and 4 seem to come together relatively higher, probably indicating a common source type. The PER of the elements of this study's toxic substances were analyzed according to Hakanson (1980). According to the analysis, the trend of *E*_*i*_ in UStD was: Cr > Ni > Cu > Pb > Zn > Cd. The average value of Ei of Cu, As, Cd, Zn, and Cr indicated a low hazard for the metals examined, hence pointing to a low PER.

According to *EF* values, Cr and Zn values smaller than 1 imply no enrichment, while Cu, Ni, and Pb values of 1–2 represent deficiency to minimal enrichment. Cd and Cr were determined to have moderate enrichment due to their values being calculated between 2 and 5. Their values represent deficiency to minimal enrichment based on their *EF* values, whereas the *EF* values showed significant enrichment for Cd and Cr. It is recognized that several anthropogenic factors contribute to the formation of HMs in UStD (Rajaram et al. 2014), including vehicle emissions from traffic and industrial emissions (Mehmood et al. 2019). For instance, Gupta et al. (2022a) expressed that toxic metals such as Mn, Zn, Cu, Pb, Cd, Cr, and Ni can be emitted into the surrounding soil in road dust particles and cause air pollution through leaching in the order of Mn (9.62%) < Cr (1.26%) < Ni (8.93%) < Cu (10.83%) < Zn (10.93%) < Pb (31.27%) < Cd (36.74%). Such activities upset the natural biogeochemical cycle of the natural ecosystem and lead to serious risks to human health (Bisht et al. 2022; Khan et al. 2023). Hence, street sediments' composition, quantity, distribution pattern, and source identification must be thoroughly assessed to accurately indicate urban environmental status (Shabbaj et al. 2018; Luo et al. 2022). All studied metals were analyzed, and the PER ranged from 117.94 to 1299.47, with a mean value of 607.43 by Gupta et al. (2022b). In comparison to the other metals, Cd contributes as high values. Therefore, the environmental contamination posed by this metal in diesel fuel, lubricants, and rubber coatings should be carefully evaluated.

### Health risk assessment

The study of UStD environmental risks and pollution factors is of major importance, giving insight into emission sources and assisting decision-making for resilient cities due to the entry of airborne suspended particulate matter in the ambient air into nearby areas (Kamani et al. 2015; Luo et al. 2019). Industrial emissions can be the main sources of anthropogenic emissions in Yenimahalle. Cd, Cr, and Cu could be partly released from fossil-fuel combustion. Table 5 shows that hazard coefficient (HQ) values, hazard index, and cancer risk results were calculated for adults: Cr, Ni, Cu, Pb, Cd, and Zn. The highest HQ_ing_ was estimated for Cd (2.04E-01) and Cu (2.67), although the lowest values were found for Cr elements (9.83E-02). These values present a low potential to cause non-carcinogenic risk (HQ < 1). The HQ_ing_ values for Ni (2.88E-02) were also high and comparable to Cd-Cu, and the Ni presented the highest risk values regarding the HQ_Inh_ (2.20E-07) and HQ_Der_ (2.99E-06), thus being recognized as the most hazardous element. The HI (Hazard Index) values were less than one, and there was no significant non-carcinogenic risk due to these HMs.

**Table 5:** Health risk assessment for adults

Elements	D_Ing_	D_Inh_	D_Der_	HQ_Ing_	HQ_Inh_	HQ_Der_	HI
Cd	2.04E-04	3.29E-10	1.09E-08	2.04E-01	5.71E-07	1.09E-03	2.06E-01
Cr	2.81E-04	5.78E-09	1.92E-07	9.83E-02	3.51E-06	1.37E-01	2.35E-01
Cu	8.24E-04	3.31E-09	1.10E-07	2.06E-02	8.29E-08	9.19E-06	2.06E-02
Ni	5.93E-04	4.53E-09	1.51E-07	2.88E-02	2.20E-07	2.99E-06	2.88E-02
Pb	1.94E-03	4.95E-09	1.65E-07	5.54E-01	2.45E-06	3.14E-04	5.55E-01
Zn	1.22E-03	9.30E-09	3.10E-07	5.91E-02	4.51E-07	6.14E-06	5.91E-02

Table 6 shows that children's non-carcinogenic health risks were analyzed for Cr, Ni, Cu, Pb, Cd, and Zn. The highest HQ_ing_ was estimated for Zn (3.70) and Pb (3.37), although the lowest values were found for Cr (5.70E-01). The other highest risk values found for Ni (1.80E + 00) and Cd (1.24E + 00) and comparable regarding the HQ_Inh_ (3.82E-07)—(5.71E-07) and HQ_Der_ (1.19E-04)—(4.67E-03), thus being recognized as the most hazardous element. The HI values were less than one, and there was no significant non-carcinogenic risk for Cr and Cu. However, Pb, Ni, Cd, and Zn showed the highest HI with a value of 3.37, 1.80, 1.25, and 1.25.

**Table 6:** Health risk assessment for children

Elements	D_Ing_	D_Inh_	D_Der_	HQ_Ing_	HQ_Inh_	HQ_Der_	HI
Cd	1.24E-03	5.71E-10	4.67E-08	1.24E + 00	5.71E-07	4.67E-03	1.25E + 00
Cr	1.88E-02	1.00E-08	8.21E-07	5.70E-01	3.51E-06	1.37E-01	7.07E-01
Cu	5.01E-03	5.75E-09	4.71E-07	1.25E-01	1.44E-07	3.92E-05	1.25E-01
Ni	3.61E-03	7.86E-09	6.44E-07	1.80E + 00	3.82E-07	1.19E-04	1.80E + 00
Pb	1.18E-02	8.59E-09	7.03E-07	3.37E + 00	2.45E-06	1.34E-03	3.37E + 00
Zn	7.41E-03	1.61E-08	1.32E-06	3.70E + 00	7.83E-07	2.45E-04	1.25E + 00

Overall, the analyses showed that HQ_ing_ had the biggest sink in the pathway exposure contribution for both population segments (Trujillo-González et al. 2016), subsequently skin contact (Urrutia-Goyes et al. 2018), and similar to some previous studies, the inhalation route (Qadeer et al. 2020; Adewumi 2022). For both population groups, HQ_ing_ is higher than HQ_der_ and HQ_inh_. With respect to the six HMs, the HQ_inh_ values for Cd and Pb are higher than the other HMs for children. Because of hand-to-mouth behavior and short stature, children are more frequently exposed to UStD as they are closer to the ground or the street (Kirel et al. 2005). Pb and Cd are poisonous metals with significant potential effects on human health (Yang et al. 2013), particularly children (Safruk et al. 2017). From the non-carcinogenic effect results, ingestion of dust particles < 90 μm in particle size can be easily re-suspended in the atmosphere and is the major exposure route to HMs in dust (Mohmand et al. 2015) as compared to inhalation and dermal absorption in particular (Doyi et al. 2020). Nevertheless, dermal exposure to Cu, Cr, Cd, Ni, and Zn particles is nearly insignificant relative to other exposure pathways. Numerous previous works on health risks related to the current situation of local communities have emphasized the assessment of concentrations, origin, particle size, spatial properties, and contamination (Chen et al. 2019). For instance, Zhang et al. (2023) conducted research to assess ecological and health pollution risks on local HMs in UStD of Baiyin City and found that Cd is the major constituent of HMs pollutants. Consequently, it can be concluded that exposure to toxic metals in UStD alone will not cause serious health hazards in the workplace. Both cancer and non-cancer risks calculated from exposure to toxic HMs from UStD, as well as the instantaneous acute intake, are under a high degree of uncertainty.

The RI values indicated a potential for carcinogenicity to the local population as a result of exposure to hazardous elements through street dust in Table 7. Ni, Cr, and Pb are among the metals contained in street dust and are classified as having a cancer-causing effect on particular human organs like kidneys, lungs, and brain (IARC). A thorough assessment of a person's lifetime carcinogenic risk has been conducted and presented. Since all carcinogenic elements are above the threshold limit, exposure to Pb, Ni, and Cr poses significant risks to adults as well as to children. These results would support decision-making aimed at reducing human health risks from metals, thereby promoting the creation of safe, affordable, and resilient cities. Further investigations are needed to validate the likelihood that elements are mainly responsible for the carcinogenic risk because the calculated values for RI in the present study were more significant than the safe range.

**Table 7:** Carcinogenic risk of HMs in SD to the population exposed

Carcinogenic metals	LADD_ing_	LADD_inh_	LADD_derm_	SF	RI_ing_	RI_inh_	RI_derm_	Total RI
Cd	6.52E + 00	8.27E-01	3.01E-02	6.30E + 00	6.02E + 01	5.21E + 00	1.90E-01	6.14E + 01
Ni	3.45E + 02	2.26E + 01	8.22E-01	4.20E + 01	1.09E + 04	9.48E + 02	3.46E + 01	1.23E + 04
Pb	4.36E + 01	4.77E + 00	1.73E-01	4.20E + 02	2.31E + 04	2.00E + 03	7.30E + 01	2.56E + 04

### Distribution of HMs

**Fig. 4:**
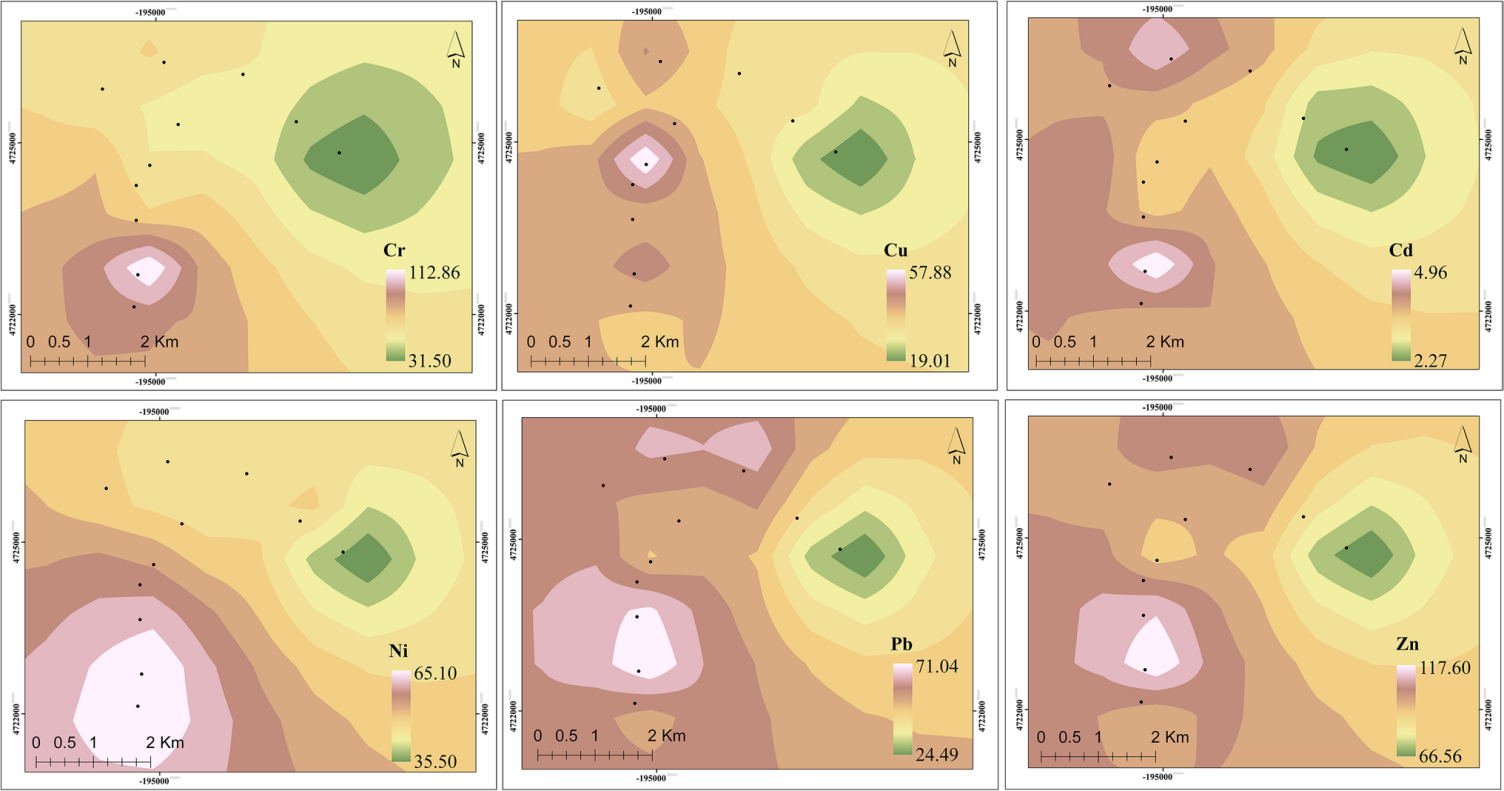
Spatial distribution patterns of (**a**): Cr, **b**: Cu, **c**: Cd, **d**: Ni, **e**: Pb, and (**f**): Zn in the UStD for different land-use types in the Yenimahalle, Ankara

Spatial distribution assessment is an assisted tool for determining the polluted and non-polluted zones in Yenimahalle on ArcGIS spatial map. In the current work site close to the local industrial site, spatial distribution patterns of Cr, Ni, Cu, Cd, Pb, and Zn in UStD are depicted in Fig. 4. To the best of our knowledge, high and medium risk zones are areas that have emerged in relatively recent years, with high population density and industrialization through the intensity and frequency of surface conditions (An et al. 2018). Given that the spatial distribution of UStD has shown that the main source of toxic metals in deposition areas is related to industry (Acosta et al. 2014; Boloorani et al. 2021), traffic, as well as the wear of tires and metal parts of motor vehicles with large-scale vertical motion (Yu et al. 2013; Men et al. 2018). Previous studies showed that the spatial distribution characteristics of HMs in the UStD around the cities (Ladonin and Mikhaylova 2020) are more polluted depending on the density of traffic and transport, agriculture, and population by Shahab et al. (2023); Peng et al. (2023) and Wang et al. (2023).

Based on the spatial distributions of Cr, Ni, Cu, Cd, Pb, and Zn, heavy traffic origin is more attributed to rise in total metal emission in comparison with similar studies (Malakootian et al. 2021; Wang et al. 2022). Skorbiłowicz et al. (2023) indicate that arithmetic averages of emitted Cr (63.59 mg∙kg^−1^), Cu (142.84 mg∙kg^−1^), Ni (17.50 mg∙kg^−1^), Zn (215.94 mg∙kg^−1^) and Pb (17.04 mg∙kg^−1^) are mostly found by wear processes of motor vehicle tires and brake pads during the dry weather period (Budai and Clement 2018). Based on content characteristics by Fan et al. (2022), three major sources could be identified: i) Co, Zn, Cu, and Pb are dominated by traffic, ii) Mn, Ni, and Cr are extracted primarily from natural sources, and iii) Hg and As are obtained mostly from coal-related industrial deposits.

## Conclusions

The present study examined environmental manner including the spatial distribution, contamination status, ecological risk assessment, and the identification of the main sources of Cr, Cd, Ni, Cu, Pb, and Zn in the UStD of Yenimahalle region. We observed hotspots of toxic metal concentration and reported results. We also compared them with those already reported from other cities. According to the conclusions, the studied area is markedly enriched by large-scale circulation. Together, the high EF for Cd and Cr in street dust represents the important levels of environmental pollution caused by these elements, which primarily come from anthropogenic sources. Cr was determined as a priority pollutant according to the assessment tool for potential eco-risks in the studied site. Based on the RI assessment, the results revealed that all parts of the Yenimahalle region have significantly high PER from industrial and metalworking-smelting emissions. This study is crucial for assessing UStD samples and characterizing the overall ecological risk of UStD due to contamination by Cr, Cd, Ni, Cu, Pb, and Zn. In general, the cancer risk for the general population was found to be very high due to the presence of Pb, Cr, and Ni crossing the threshold level (10^–6^ to 10^–4^). More studies are also needed to characterize potential hazards in Ankara City that have not been described in the contaminated UStD. Management strategies should be applied to reduce the discharge of potentially toxic metals from the industrial area. Responsible bodies such as environmental agencies and agricultural organizations should regulate the irregular practices of small industries. Particulates can also be carried into water bodies (e.g., groundwater and surface water) via urban storm flows. Consequently, future research should also investigate the effects of metals in facilitating sustainable urban development on water resources, plant uptake, and human health.

## Data Availability

The data that support the findings of this study are available from the corresponding author, upon reasonable request.
